# The role of glycogen synthase kinase 3 (GSK3) in cancer with emphasis on ovarian cancer development and progression: A comprehensive review

**DOI:** 10.17305/bjbms.2020.5036

**Published:** 2021-02

**Authors:** Mislav Glibo, Alan Serman, Valentina Karin-Kujundzic, Ivanka Bekavac Vlatkovic, Berivoj Miskovic, Semir Vranic, Ljiljana Serman

**Affiliations:** 1 Department of Biology, School of Medicine, University of Zagreb, Zagreb, Croatia; 2 Centre of Excellence in Reproductive and Regenerative Medicine, University of Zagreb School of Medicine, Zagreb, Croatia; 3 Department of Obstetrics and Gynecology, School of Medicine, University of Zagreb, Zagreb, Croatia; 4 Clinic of Obstetrics and Gynecology, Clinical Hospital “Sveti Duh”, Zagreb, Croatia; 5 College of Medicine, QU Health, Qatar University, Doha, Qatar

**Keywords:** GSK3, ovarian cancer, therapeutic target

## Abstract

Glycogen synthase kinase 3 (GSK3) is a monomeric serine-threonine kinase discovered in 1980 in a rat skeletal muscle. It has been involved in various cellular processes including embryogenesis, immune response, inflammation, apoptosis, autophagy, wound healing, neurodegeneration, and carcinogenesis. GSK3 exists in two different isoforms, GSK3α and GSK3β, both containing seven antiparallel beta-plates, a short linking part and an alpha helix, but coded by different genes and variously expressed in human tissues. In the current review, we comprehensively appraise the current literature on the role of GSK3 in various cancers with emphasis on ovarian carcinoma. Our findings indicate that the role of GSK3 in ovarian cancer development cannot be decisively determined as the currently available data support both prooncogenic and tumor-suppressive effects. Likewise, the clinical impact of GSK3 expression on ovarian cancer patients and its potential therapeutic implications are also limited. Further studies are needed to fully elucidate the pathophysiological and clinical implications of GSK3 activity in ovarian cancer.

## INTRODUCTION

According to the European Union (EU) statistics, almost 30,000 women died due to ovarian cancer throughout the Union in 2018. That accounts for 5% of all cancer-related deaths among women in the EU [1]. Because of lack of early symptoms and effective screening options [2], ovarian cancer is often diagnosed at advanced FIGO stages [3] and is responsible for the highest mortality rates among gynecologic malignancies, including breast cancer. Therefore, there is an unmet need to comprehensively study the molecular background of this disease in order to devise and institute targeted therapy.

Glycogen synthase kinase 3 (GSK3) is a monomeric serine-threonine kinase discovered in 1980 in a rat skeletal muscle. Although numbered 3, it is the only enzyme that has been proven to phosphorylate glycogen-synthase, whereas the enzymes previously being called GSK1 and 2 do not have that characteristic [4]. GSK3 has been found to play an important role in various cellular processes, including embryonic development, immune response, inflammation, apoptosis, autophagy, wound healing, neurodegeneration, and carcinogenesis [5-9]. GSK3 has two isoforms, GSK3α and GSK3β, both containing seven antiparallel beta-plates, a short linking part and an alpha helix, but coded by different genes and variously expressed in human tissues. GSK3β is coded by *GSK3B* gene on the long arm of chromosome 3 [10] and is more abundantly expressed in natural killer (NK) cells, bone marrow granulocytes, and ovaries [11]. Its molecular mass is slightly lower than that of GSK3α, as it does not contain glycine-rich N-terminal domain. It is located in both the nucleus and cytoplasm [12]. Expression of one isoform cannot compensate for the loss of another, which is especially important during embryonic development, when the loss of *GSK3B* gene is lethal [13].

**FIGURE 1:**
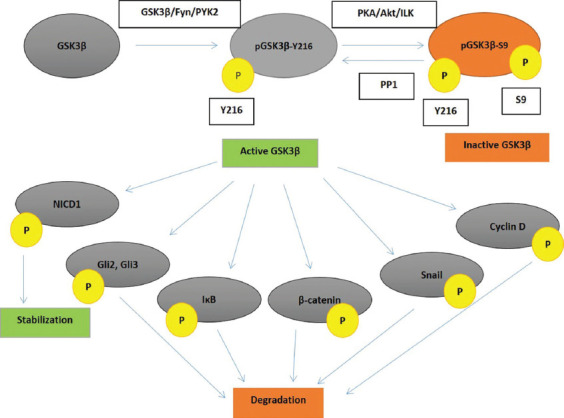
GSK3β activation, inactivation, and activity towards its key substrates. GSK3β – glycogen synthase kinase 3β; pGSK3β-Y216 – glycogen synthase kinase 3β phosphorylated at tyrosine 216; pGSK3β-S9 – glycogen synthase kinase 3β phosphorylated at serine 9; Y216 – tyrosine 216; S9 – serine 9; PKA – protein kinase A; Akt – protein kinase B; ILK – integrin-linked kinase; PP1 – protein-phosphatase 1; NICD1 – Notch 1 intracellular domain; Gli – glioma-associated oncogene; IκB – inhibitor κB; P – phosphate group; PYK2 – tyrosine protein kinase 2.

Phosphorylation of tyrosine 216 (Y216) is responsible for the constitutive activity of GSK3β [14], which is autophosphorylation during the translation process [15]. Peculiarly, GSK3β is a tyrosine-kinase during its formation but later assumes the function of a serine-threonine kinase [16]. Other kinases, such as PYK2 and Fyn, as well as proapoptotic signals, can also complete that phosphorylation [17-19]. Although constitutively active, priming phosphorylation by other kinases is usually required for GSK3β substrates [20]. On the other hand, phosphorylation of serine 9 (S9) of the GSK3β blocks the substrate binding and inactivates the enzyme [14]. Many kinases are involved in that process. Protein-kinase A, activated by cyclic adenosine monophosphate (cAMP), Akt (protein-kinase B, PKB), activated through the PIP3/mTOR pathway, and integrin-linked kinase (ILK) are the main factors performing S9 phosphorylation. Phosphorylations of some other sites that also inhibit the enzyme activity (by extracellular signal-regulated kinase [ERK], for example) [21], as well as dephosphorylations of S9 by protein phosphatases that resume the catalytic activity [22], are possible too (Figure 1).

GSK3β participates in many cellular signaling pathways (Figure 1). It often phosphorylates more than one component of a pathway and has pleiotropic, often opposite, effects on that same pathway and cell proliferation.

Within the Wnt signaling pathway, GSK3 is in a protein complex with casein kinase 1 (CK1), axin, and adenomatous polyposis coli (APC). It normally phosphorylates β-catenin, having previously been phosphorylated by CK1, which is consequently ubiquitinated and degraded in the proteasome [23]. Upon Wnt pathway activation, the protein complex GSK3/CK1/axin/APC is disintegrated, and unphosphorylated β-catenin is translocated into the nucleus, where it activates the transcription of various protooncogenes, such as *MYC*, *JUN*, cyclins, matrix-metalloproteinase, and *MDR1*, which encodes p-glycoprotein, a transporter responsible for chemotherapy resistance [23]. It seems that GSK3 activity towards β-catenin does not depend on its phosphorylation status on S9, which appears to be a protective mechanism for this important pathway when GSK3 is aberrantly phosphorylated by some kinase [24,25]. For instance, highly active Akt does not fully inhibit GSK3β activity in pancreatic and colon cancer cells [26,27]. However, a more recent study on human colorectal cancer cell lines indicates that hyperactive Akt causes GSK3β inhibition and β-catenin accumulation [28]. Apart from phosphorylating β-catenin, GSK3 can phosphorylate low density lipoprotein receptor-related protein (LRP) molecule and thus reveal a binding site for axin on LRP, which mimics pathway activation by a Wnt ligand [29].

Full-length Gli2 and Gli3 are the key GSK3 substrates among the components of the Hedgehog signaling pathway [30]. Upon phosphorylation, Gli2 is usually completely degraded in the proteasome, whereas Gli3 is processed into a transcription repressor [31,32]. In a case of GSK3β inactivation, full-length Gli proteins are activated by other kinases and translocated into the nucleus, where they activate transcription of the Hedgehog pathway components genes *GLI1* (which in turn acts as a transcription activator independent on inactivation by GSK3) and *PTCH*, protooncogenes *IGF2* and *MYC*, as well as apoptosis inhibitor *BCL2* [13]. Besides that, by phosphorylating suppressor of fused homolog (SuFu), GSK3β reduces SuFu’s potential to bind to Gli, which in turn accumulates in the nucleus and activates transcription [33]. Despite the traditional view of GSK3β being inactivated by S9 phosphorylation, recent studies by Trnski et al. suggest that, regarding phosphorylation of Gli3, pGSK3β-S9 is actually an active and pGSK3β-Y216 an inactive form of GSK3β [34]. Furthermore, treating colon cancer cells (both cell lines and *ex vivo* specimens) with GSK3β inhibitor lithium chloride (LiCl) enhances its phosphorylation on S9 and promotes the formation of Gli3/SuFu/GSK3β complex, with consequent phosphorylation and inactivation of Gli3. In contrast, GSK3 knockdown in cells causes Hedgehog pathway activation [34], which indicates that pGSK3β-S9 is not necessarily inactive.

In the NFkB signaling pathway, GSK3 activates a kinase cascade resulting in phosphorylation of inhibitory IkB and its dissolution from nuclear factor-kB (NFkB) [35], which is translocated into the nucleus and activates transcription. The effect is the same when GSK3 phosphorylates inhibitory p100 molecule in multiple myeloma cells [36]. On the other hand, phosphorylation of p65 in HeLa cervical cancer cells [37] and IkB kinase (IKK) inhibition together with IkB stabilization in human astrocytes [38] makes GSK3β also a negative regulator of this pathway.

Notch, a pathway usually associated with suppression of cell differentiation [39], is activated by the detachment of its intracellular domain (Notch intracellular domain – NICD) and its nuclear translocation. GSK3β phosphorylation in Ser/Thr – Pro – Ser/Thr regions stabilizes NCID in Notch 1 [40,41] and Notch 3 [42], and inactivates it in Notch2 [43]. Mitigating Notch1 recycling is likewise an example of negative regulation of this pathway by GSK3β [44].

In response to transforming growth factor beta (TGF-β), ERK kinase inactivates GSK3β, resulting in Snail protein accumulation [45,46] and promotion of epithelial-mesenchymal transition (EMT), which is characterized by the loss of cell polarity and serves as a pre-condition for tumor invasion and metastasis [11]. In contrast, GSK3 decreases cell susceptibility to TGF signaling by phosphorylating Smad3 [47].

Interestingly, GSK3 may act as both a proapoptotic and an antiapoptotic factor, depending on cell type and signaling environment [13]. GSK3β promotes apoptosis in conditions of hypoxia [48] and DNA damage [49] by inhibiting cell survival signals like cAMP-response element binding (CREB) protein and heat-shock protein 1 [50] and activating proapoptotic transcription factors such as p53 [49]. The relationship between GSK3 and p53 is complex. Phosphorylation of p53 (previously phosphorylated by DNA-protein-kinase) by GSK3β blocks its interactions with inhibitory mouse double minute 2 homolog (MDM2), thus contributing to the stabilization of p53 and its activity as a transcriptional activator of proapoptotic genes [51]. In addition, activity of GSK3β increases in interactions with p53 by a phosphorylation independent mechanism [49]. Moreover, inhibition of GSK3β is shown to promote GSK3β, MDM2, and p53 sequestration in the cytoplasm, where p53 cannot act as a transcriptional activator [52]. However, inhibition of GSK3β can lead to MDM2 hypophosphorylation, p53 accumulation and consequent apoptosis, which suggests an antiapoptotic activity of GSK3β. By phosphorylating Bax, GSK3β localizes it to mitochondria and induces its proapoptotic activity [53,54]; however, GSK3 inhibition also promotes apoptosis, by hypophosphorylation, and thus decreasing activity, of apoptosis regulator Bcl2 [55,56].

The effects of GSK3 on the process of autophagy are more straightforward as GSK3 opposes it not only by promoting glucose metabolism and utilization, but also by phosphorylating protein raptor (regulatory associated protein of mTOR), which activates mTORC1 complex and inhibits autophagy [57].

GSK3 is actively involved in cell cycle regulation since the key factors (cyclins, cyclin-dependent kinases, key points regulators, and transcription factors) are among its substrates [13,58]. GSK3 phosphorylates cyclins D and E, important for transition from G1 to S phase, and causes their degradation [59,60]. Transcription factors c-myc and c-fos (also S phase promoters), which are phosphorylated primarily by dual-specificity tyrosine phosphorylation-regulated kinase (DYRK), are also phosphorylated by GSK3β, which guides them toward degradation [61]. On the other hand, GSK3β inhibition in pancreatic cancer cells suppresses Rb phosphorylation by cyclin D/cyclin-dependent kinase 4 or 6 complex and its subsequent degradation [62].

EMT is characterized by the loss of functional E-cadherin (encoded by the *CDH1* gene). As already mentioned, GSK3 phosphorylates and inhibits Snail protein, the *CDH1* gene transcription repressor, thus indirectly increasing E-cadherin expression [63]. GSK3β also contributes to this by the degradation of β-catenin that is bound to E-cadherin [23]. GSK3β takes part in decreasing matrix-metalloproteinase expression and is therefore thought to maintain a stem cell phenotype in tumor cells, but without diminishing their metastatic potential [64].

The role of GSK3 in tumor formation and promotion is controversial, as it has diverse effects on numerous cellular processes and pathways, which can also differ among different cell types. There is a clear evidence for both prooncogenic and tumor-suppressive effects of GSK3. Several illustrative examples will be briefly described here, although the list should not be considered exhaustive.

In glioblastoma multiforme, the most common and most lethal brain tumor, GSK3 inhibitors facilitate apoptosis through inhibition of antiapoptotic mechanisms in mitochondria and inhibit the NFkB pathway that is essential for cell survival [56,65]. GSK3β also seems to be responsible for NFkB aberrant activity in pediatric acute lymphoblastic leukemia [66] and pancreatic cancer [67] cells. In renal cancer cells, GSK3 inhibitors induce cell cycle arrest, differentiation of the malignant cells, and autophagy [68].

In contrast to the neoplasms mentioned above, in skin [69], oral [70], and lung [71] cancers a high expression of inactive pGSK3β-S9 is found, which suggests tumor-suppressing effects of the enzyme in those cancers. In melanoma, microRNA miR-769 inhibits GSK3β activity during the tumor development process, which also indicates tumor-suppressing effects of GSK3 [72].

## GSK3 IN OVARIAN CANCER DEVELOPMENT

Recognition that GSK3 is a factor in ovarian cancer development dates back to the late 1990s, when the analysis of 61 ovarian cancer samples of different histologic types in Japan revealed that 33% of endometrioid and 14% of mucinous carcinoma specimens had β-catenin mutations changing serine and threonine residues targeted by GSK3. The patients with these mutations had more abundant nuclear β-catenin expression and were on average 10 years younger than the mean age of all included patients [73]. Rask et al. (2003) were the first to discover GSK3β overexpression in ovarian cancer cells [74] (Table 1), with increased expression of β-catenin and lower expression of APC also described. In ovarian cancer cell lines, a nuclear localization of β-catenin has been reported, which is not the case in ovarian cancer tissue samples. That was explained by E-cadherin overexpression, which binds β-catenin into a perimembranous protein complex, which raised the question of β-catenin being included in ovarian cancer development as a transcription activator, cellular integrity stabilizer or both [74]. Further investigations by Usongo et al. revealed a perimembranous expression of β-catenin in normal ovarian epithelial cells, in contrast to those stimulated by Wnt3 or by GSK3 inhibitor LiCl, in which β-catenin was expressed in the cell nucleus. However, despite nuclear localization of β-catenin, its target genes’ transcription is not elevated [75].

**TABLE 1:**
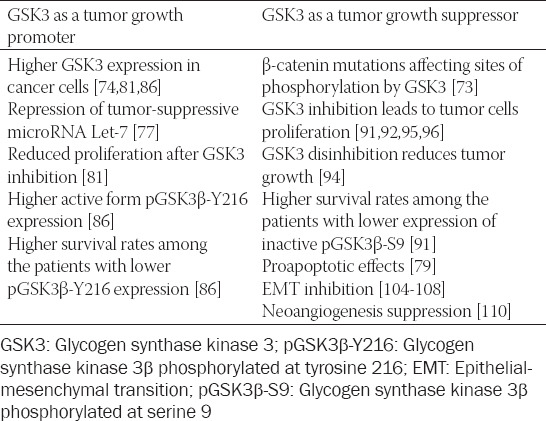
A summary of various GSK3 effects on ovarian cancer cell growth

Investigations pertaining to the Hedgehog and p53 pathways have been less extensive. However, it has been found that GSK3 inhibition reduces expression of activating Gli3A and PTCH (Hedgehog pathway activity indicator) [76]. It has also been found that GSK3β reduces the expression of tumor-suppressor microRNA Let-7 in ovarian cancer cells, and it seems that p53 is involved in the process. In contrast to the expectations, p53 expression is lower when GSK3β is active and increases after GSK3β inactivation [77]. A possible explanation is that damaged protein glycosylation in cancer cells causes accumulation of those proteins and subsequent endoplasmic reticulum stress. That condition, by a still unknown mechanism, facilitates GSK3-mediated phosphorylation of p53 at serine 376, which acts as a degradation signal [78]. On the other hand, investigations of resveratrol as an apoptotic inducer through GSK3 activation, N-glycosylation suppression, and endoplasmic reticulum stress suggest a proapoptotic role of GSK3 and endoplasmic reticulum stress [79].

Wulfkuhle et al. found that GSK3β expression varies more between the specimens of the same histologic type than among different ovarian cancer histologic types, which made them emphasize an individual approach to every patient [80]. Cao et al. found a higher immunohistochemical expression of GSK3β in ovarian cancer cells, whereas expression of the enzyme phosphorylated on serine 9 (pGSK3β-S9) varied among two cell lines included in the study. The treatment with LiCl increased pGSK3β-S9 expression, while overall expression remained unchanged. The treatment with LiCl also caused a decrease in cell number, which led to the conclusion that GSK3β promotes ovarian cancer cell proliferation and tumor growth (Table 1) [81]. Cyclin D levels were also considerably lower after GSK3β inhibition, which was in contrast to the common belief that GSK3β phosphorylates cyclin D and facilitates its degradation [81]. The importance of cyclin D lies in the fact that it can induce cell cycle arrest even when cyclin E is constitutively active [82]. Cao et al. therefore suggest that GSK3β is the key driving force in ovarian cancer development, the explanation being its metabolic effects, namely decreased glycogenesis and increased glucose utilization through the process of glycolysis [81]. This view is supported by the facts that cancer cells have higher metabolic needs [83] and depend heavily on glycolysis [84]. The authors also emphasize the prooncogenic effects of GSK3β on the NFkB pathway [81], although it has previously been said that those effects could be tumor suppressive as well. Glycolysis hypothesis is also supported by the findings that glycolysis inhibitor apatinib (which also has other effects such as vascular endothelial growth factor [VEGF] receptor 2 inhibition, Akt inhibition, and GSK3 reactivation) significantly reduces ovarian cancer masses both *in vitro* and in mouse transplants *in vivo* [85]. Fu et al. confirmed GSK3β overexpression in ovarian cancer cells and found that active form pGSK3β-Y216 is also highly expressed [86], which again favors prooncogenic role of the enzyme. Furthermore, higher GSK3β levels are associated with advanced FIGO stages and elevated CA-125 levels [86]. Overexpression of pGSK3β-Y216 correlates not only with FIGO stage and CA-125 levels but also with the residual tumor masses and unfavorable chemotherapy outcomes. The survival analysis revealed that patients with lower levels of GSK3β and pGSK3β-Y216 had significantly higher overall survival. Finally, GSK3β inhibition leads to tumor growth retardation both *in vitro* and *in vivo*: whereas tumor masses in mice were significantly lower throughout the entire experiment, tumor volumes were significantly lower during most of the study, but not at the very end [86].

Findings about GSK3 overexpression and hyperactivity prompted further research on its upstream regulation, mainly protein kinase B (PKB, Akt), which is believed to phosphorylate GSK3β on S9 and cause its inactivation. Do et al. studied activin A, peptide hormone that participates in follicle development and FSH β-chain expression regulation [87,88]. It can act in both Smad-dependent (like TGF-β) and Smad-independent (through mitogen-activated protein [MAP] and phosphatidylinositol-3,4,5-trisphosphate [PIP3] kinases) way [89]. Activin A has no effect on normal ovarian cell proliferation but can boost cancer cell proliferation [90]. Do et al. showed that activin A activates Akt, which in turn phosphorylates GSK3β on serine 9 and inactivates it to a greater extent than GSK3α does. Immunohistochemical expression of pGSK3β-S9 differs depending on tumor malignant potential. Thus, in benign ovarian neoplasms, pGSK3β-S9 expression is predominantly localized (polarized) around apical gap junctions, whereas in malignant neoplasms (carcinomas) its expression is diffuse. Borderline tumors have mixed expression, both polarized and diffuse. Inactivation status of GSK3β is associated with survival, i.e., patients with lower levels of inactivated GSK3β live longer (Table 1) [91]. This study supports suppressive effects of GSK3β on ovarian cancer development, and similar findings were reported by Cianfrocca et al. when studying endothelin-1 effects. Apart from GSK3β phosphorylation through the cascade endothelin-1 receptor → β-arrestin → PIP3 kinase → ILK → Akt → GSK3β, it was shown that endothelin-1 receptor activation facilitates removal of GSK3β from the complex that phosphorylates β-catenin, which leads to its degradation [92]. Oral hypoglycemic drug metformin reduces ovarian cancer cells growth in Akt-depending manner. It activates AMP kinase [93], which phosphorylates and inhibits Akt, leading to GSK3β disinhibition. Cyclin D is then phosphorylated by GSK3β and degraded, resulting in cell cycle arrest [94]. Polyphenol resveratrol, apart from causing endoplasmic reticulum stress that has already been mentioned, inhibits both Akt and ERK, thus facilitating GSK3β reactivation on OVCAR3 cell line, with a consequent lower expression of cyclin D1 and reduced cancer cell number [95]. Other Akt inhibitors, such as GSK690693, GSK2141795, GDC-0068, MK-2206, AKT-IN-1 and AKT-IN-2, also have the potential to inhibit the growth of ovarian tumors transplanted into mice and some of them are being studied in clinical trials [96].

## GSK3 IN EPITHELIAL-MESENCHYMAL TRANSITION, NEOANGIOGENESIS, AND METASTASIS

Due to their mesothelial origin, ovarian surface cells normally express mesenchymal N-cadherin, rather than epithelial E-cadherin [97]. Once affected by metaplasia, they start expressing E-cadherin, which can promote the EMT in ovarian surface cells [98]. Early carcinoma cells express E-cadherin, whereas advanced carcinoma cells, and particularly metastases, are characterized by low E-cadherin expression [99], which suggests that a loss of E-cadherin is a pre-condition for metastasis. Apart from β-catenin, it is thought that Snail protein plays a key role in EMT induction. Indeed, patients with higher Snail expressions have significantly shorter overall survivals [100]. It appears that the TGF-β and Wnt pathways, activated alone, promote EMT; however, when activated together, those pathways actually suppress EMT [101]. Moreover, GSK3β inhibitor concentration affects β-catenin level, which in turn decides the destiny of EMT. Lower doses of LiCl and lower β-catenin levels suppress EMT, while higher doses of LiCl and higher β-catenin concentrations have the opposite effects [101].

Endothelin receptor activation, besides its effects on proliferation, also promotes EMT due to decreased β-catenin and Snail degradation after Akt activation and GSK3 inactivation. In that study, E-cadherin levels were lower and N-cadherin expression reappeared [102]. Forkhead box protein C2 (FOXC2) has similar effects by activating Akt and/or ERK in platinum-resistant ovarian cancer cell lines [103]. The role of GSK3β in EMT suppression is strongly supported by the fact that compounds that reduce serine 9 phosphorylation of the enzyme (like emodin, anthraquinone derived from rhubarb and aloe, and sPSB3 molecule) inhibit EMT in ovarian cancer cell lines (Table 1) [104-106]. Several microRNAs, such as miR16 and miR203a-3p, may exhibit similar effects, although their precise mechanisms are not known yet [107,108].

The study of Burkhalter et al. showed that integrins α2, α3, and β1 have an important and complex role in EMT and β-catenin levels control: they reduce membranous E-cadherin expression, probably by E-cadherin rearrangement, as they do not reduce the overall expression of E-cadherin. That reduction promotes EMT. Also, β-catenin liberated from the complex with E-cadherin is translocated into the nucleus, where it promotes gene transcription, including those coding for matrix-metalloproteinase, which again promotes EMT and may account for Wnt hyperactivity even without genetic or epigenetic alterations of its components. On top of that, b1 integrin activates ILK kinase that inhibits GSK3β, which leads to further accumulation of β-catenin [109].

When oxygen supply is normal, GSK3β suppresses neoangiogenesis by phosphorylating hypoxia-inducible factor 1 (HIF1), which increases its affinity towards ubiquitin-ligase and its subsequent ubiquitination and degradation. In hypoxia, however, GSK3β activity decreases and stabilized HIF1 promotes the *VEGF* gene transcription [110]. VEGF then induces neoangiogenesis and affects neighboring cells by activating Akt and further inhibiting GSK3β [111].

## THE ROLE OF GSK3 IN CHEMOTHERAPY RESISTANCE

### Vincristine

A repeated exposure of SKOV3 cell line (metastatic ovarian adenocarcinoma) to vincristine revealed a subpopulation of cells resistant to vincristine. The analysis of those cells showed that the resistant cells had increased expression of Wnt family member 5a (Wnt5a), survivin, and β-catenin and elevated phosphorylation of Akt and GSK3β (on serine 9). *WNT5A* gene silencing, as well as PIP3 kinase inhibition, succeeded to reduce survivin and β-catenin levels and Akt and GSK3β phosphorylation. Therefore, it seems that aberrantly active Wnt pathway favors vincristine resistance through GSK3β inhibition [112].

### Paclitaxel

In ovarian cancer cells resistant to paclitaxel, an increased expression of Dishevelled protein, a positive regulator of Wnt pathway, was observed. Downstream of Dishevelled, S9 phosphorylation of GSK3β and β-catenin levels were increased, as were the expression of its target genes, such as *MDR1* and *BCL2*, which seems to be the key features of resistance to paclitaxel. Akt is again involved in GSK3β inhibition, as Akt inhibitors were able to restore GSK3β activity [113]. Another research group reported four times higher GSK3 expression in resistant cells. Since SKOV3 cells used in the experiment normally do not express GSK3α, it appears that at least part of that increase was due to GSK3β [114]. However, expressions of phosphorylated forms were not assessed, and it is therefore possible that the expression was elevated due to S9 phosphorylated, inactive, form.

### Cisplatin

Ovarian cancer cells resistant to cisplatin had similar overall expression of GSK3β and of pGSK3β-Y216, but significantly lower expression of silenced pGSK3β-S9 in comparison to the source cell line. The protective role of GSK3β toward cisplatin resistance is additionally supported by the facts that the cells treated with LiCl have lower cell death rates when exposed to cisplatin, that inhibitory concentration 50 (IC50) of cisplatin for those cells is significantly higher, and that those two findings are absent in cells with constitutively active pGSK3β-S9A, not prone to serine 9 phosphorylation [115]. Tangeretin, a flavonoid from citrus fruit, succeeded to restore cisplatin sensitivity to resistant cells, but the effect was 60% lower if the cells were pre-treated with GSK3β inhibitor LiCl [116]. Another study revealed a substantially lower GSK3β activity in cisplatin resistant cells compared with the source cell line. Akt activities were similar, but ERK activities were elevated in cisplatin-resistant cells [117].

## GSK3 AS A PROGNOSTIC FACTOR

The clinical data on the prognostic role of GSK3 in ovarian cancer patients are currently limited. Two studies that analyzed survival related to GSK3 status have already been presented above: Fu et al. found higher survival rates among the patients with lower GSK3β and active pGSK3β-Y216 expression, whereas Do et al. claimed survival rates to be higher among the patients with lower expression of inactive pGSK3β-S9 [86,91]. The third study, conducted by Shin et al. and restricted only to serous ovarian carcinomas, found that among patients with higher GSK3β activity there were fewer at advanced FIGO stages. However, the results did not reach the significance level [5]. The same study found that, in contrast to GSK3β expression, DNA-protein kinase, Akt3, and p53 overexpression clearly correlated with shorter survival [5].

## GSK3 AS A POTENTIAL THERAPEUTIC TARGET

Since the exact function of GSK3 in ovarian cancer development is still unclear, both its activation and inhibition have been tried as therapeutic options.

### Activation of GSK3 as a therapeutic option

The study in which metformin-induced AMP kinase activation caused Akt inhibition and GSK3β restitution, with subsequent cyclin D degradation and cell cycle arrest, has already been discussed above [94]. Regardless of mechanism, several studies found a positive effect of metformin on survival of ovarian cancer patients [118,119], which was confirmed in a recently published meta-analysis study [120].

Akt inhibitors disinhibit GSK3, and a number of them have been investigated in clinical studies for their therapeutic potential (Tables 2 and 3).

**TABLE 2:**
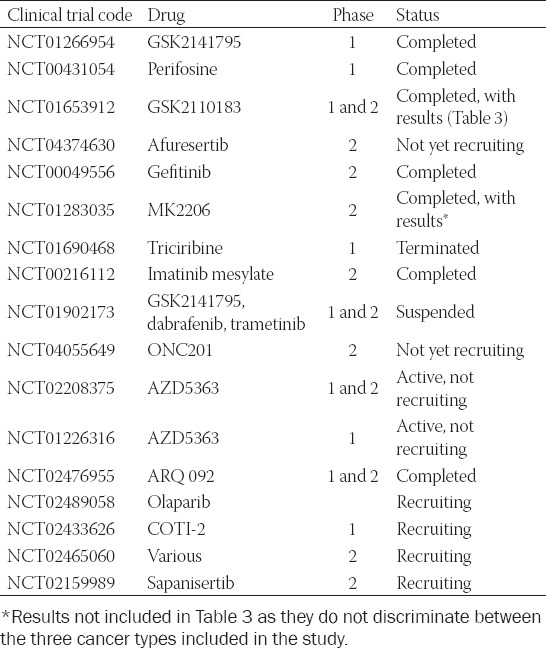
Akt inhibitors in current clinical trials for ovarian cancer treatment (https://clinicaltrials.gov/ct2/results?cond=Ovarian+Cancer+&term=Akt&cntry=&state=& city=&dist=)

**TABLE 3:**
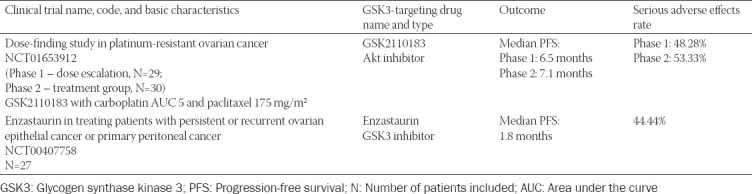
GSK3-targeted ovarian cancer therapy – clinical trials with published results

In addition to conventional drugs, some phytochemicals and Chinese traditional medicine products have also been investigated. Thus, fungus *Trametes robiniophila* (Chinese: Huaier) and Fuling granules (mixture of *Aconitum napellus*, *Wolfiporia extensa*, *Patrinia heterophylla*, and the root of *Paeonia rubra*), both traditional Chinese medicines, act as Akt inhibitors, reduce β-catenin expression, and induce apoptosis. Fuling granules, in addition, inhibit the TGF-β pathway and suppress EMT. Polyphenol resveratrol, a red wine component, which is an anti-inflammatory agent and protects against oxidative stress, has various effects on tumor mass reduction [121]. It can inhibit Akt and ERK and restore GSK3β activity in human ovarian cancer cell lines *in vitro* [95] and induce apoptosis of malignant cells [79].

### Inhibition of GSK3 as a therapeutic option

Several studies took the opposite approach – to inhibit GSK3 when it is overactive.

LiCl, already discussed in this review, inhibits GSK3 in two ways: it competes with magnesium ions for the binding site on the enzyme and, as its charge is 1+ compared to magnesium’s 2+, changes charge density around the enzyme [122]. LiCl also favors S9 phosphorylation of GSK3β [123]. In contrast to Cao et al., who found reduced cell number and reduced cyclin D expression after exposure to LiCl, as well as reduced tumor mass *in vivo*, more recent study by Novetsky et al. found a minimal activity of LiCl toward tumor mass reduction and insignificant effects of LiCl when combined with paclitaxel or cisplatin. According to their results, only tumor cells’ metabolism reduction was significant and only after the exposure of cells to 10 mM LiCl (the concentration used by Cao et al. as well), whereas 1 mM LiCl had no effect at all [81,124].

Compound 9ING41 also fosters S9 phosphorylation but acts also as a competitive inhibitor at the GSK3 enzyme ATP binding site. An increased cyclin D1 expression was seen in 9ING41-treated SKOV3 cells, which is consistent with the hypothesis that GSK3β inhibition leads to accumulation of cyclin D1. Surprisingly, however, the proliferation rate of those cells was not elevated, and an increase in apoptosis was seen. 9ING41 treatment reduced tumor masses in mice with transplanted human ovarian cancer cells and no adverse effects were noticed. LiCl in this experiment failed to reduce tumor masses *in vivo* [125]. There is a clinical trial currently recruiting participants that aims to assess 9ING41 activity toward advanced cancers, including ovarian cancer: (https://clinicaltrials.gov/ct2/show/record/NCT03678883?term=GSK-3%CE%B2&cond=Ovarian+Cancer&draw=2&rank=1).

(2*Z*,3*E*)-6-bromoindirubine-3’-oxime (BIO) is a competitive GSK3β inhibitor at ATP binding site that proved to be efficient in a proliferation suppression and cancer cell number reduction in several cancer cell lines, such as breast, pancreatic, osteosarcoma, and melanoma lines [126-129]. Yu and Zhao showed that BIO exhibits the same effects on ovarian cancer cell lines, along with reduced number of cells in S and M-phase and reduced number of invasive and migrating cells. The mechanisms that can account for these effects are decreased expressions of GSK3β, β-catenin, matrix-metalloproteinase, and their corresponding mRNA molecules [130].

AZD1080, another ATP binding site inhibitor, reduces the expressions of GSK3β, cyclin D1, cyclin-dependent kinases 1 and 2, matrix-metalloproteinase 9 and antiapoptotic molecule B-cell lymphoma-extra large (Bcl-xL), and blocks proliferation, filopodia production, invasiveness, and metastasis of ovarian cancer cell lines [131].

Bearing in mind that a competitive inhibition may be overridden by increased substrate concentrations, Gao et al. developed a series of benzothiazinone uncompetitive (allosteric) GSK3β inhibitors, the majority of which expressed moderate antiproliferative activity on ovarian cancer cell lines. Their tumor-suppressive activity on A2780 and OVCA433 lines differed depending on the numbers of carbon atoms in their structures, the compound named 20 g being most specific towards GSK3β and most effective in suppressing malignant cell growth. Its effects were confirmed *in vivo* in mice with transplanted human cancer cells [132].

Ursolic acid from *Oldenlandia diffusa* induces apoptosis in leukemia, melanoma, prostatic, breast, and colon cancer cells by affecting various signaling pathways (Akt, NFkB, ERK, and c-Jun) and oxygen reactive species concentrations [133-136]. In SKOV3 and A2780 cells, ursolic acid enhances S9 phosphorylation of GSK3β and its inactivation, β-catenin stabilization and accumulation and, surprisingly, reduces expression of cell survival signals (c-myc and Bcl-xL) and matrix-metalloproteinase, which in turn facilitates apoptosis and reduces tumor invasiveness [137].

GSK3β inhibitor enzastaurin entered phase two clinical trials in three independent studies. The results of one of these studies have been published: 27 ovarian cancer patients were treated with enzastaurin, having a median overall survival of 15.1 months and median progression-free survival of just 1.8 months, whereas 12 patients experienced serious adverse effects (https://clinicaltrials.gov/ct2/show/results/NCT00407758?term=enzastaurin&cond =Ovarian+Cancer&draw= 2&rank=2; Table 3).

## DISCUSION AND CONCLUSION

The role of GSK3 in ovarian cancer development cannot be decisively determined as the currently available data support both prooncogenic and tumor-suppressive effects (summarized in Table 1).

To elucidate the exact role of GSK3 in ovarian cancer development further research is necessary. In ovarian cancer cells, the impact of GSK3 on some signaling pathways (e.g., the Notch pathway) has not been studied yet, which is important because GSK3 has different effects in different cells and under different circumstances. Also, signaling pathways have been usually examined individually and separately from other pathways, thus losing the opportunity to get a broader insight into the pleiotropic effects of GSK3 and potential crosstalk between various pathways at the level of GSK3 [138].

The studies concerning GSK3 expression in ovarian cancer cells most commonly report its overexpression, independently on the cell lines used. One study also assessed the expression of the active form pGSK3β-Y216 and found its overexpression as well, which led to the conclusion that GSK3β is both overexpressed and overactivated in ovarian cancer cells [86]. However, the expression of the inactive form pGSK3β-S9 was not measured in these experiments although it can possibly account for the overexpression. Despite that, and despite the fact that overexpression does not necessarily mean overactivation, these studies clearly propose GSK3 hyperactivity in ovarian cancer cells and its role as a driving force in cancer development [74,81,86].

On the other hand, the studies concerning the upstream control of GSK3 activity, especially Akt kinase, suggest that GSK3 is a tumor growth suppressor because activated Akt inhibits GSK3 and β-catenin subsequently accumulates. These findings are supported by the fact that after Akt inhibition, GSK3 activity is reinstated and proliferation slows down [94]. In this group, the results are also consistent independently of the cell line used. However, aberrant pathway activity may be a result of the mutations of its components, regardless of GSK3 activity. For example, *CTNNB1* gene mutations targeting sites phosphorylated by GSK3 on β-catenin [73], mutations of positive pathway regulators that cause its constitutive activity (such as Frizzled), mutations or epigenetic silencing of negative regulators, and mutations of proteins in a complex with GSK3 may all cause Wnt pathway hyperactivity [139]. Besides, β-catenin levels are controlled not only by GSK3 but also by E-cadherin levels [109].

Taking into account these opposite findings, it is surprising that only the study of cisplatin resistance by Cai et al. assessed the expression of both pGSK3β-S9 and pGSK3β-Y216 on the same specimens, the only difference between the cells sensitive and resistant to platinum being elevated expression of inactive pGSK3β-S9 in the subpopulation of resistant cells [115].

A more detailed mechanism of S9 phosphorylation suggests that taking pGSK3β-S9 as an active and pGSK3β-Y216 as an inactive form might be oversimplified. Phosphate from S9 lies in the site that recognizes the phosphate group of the pre-phosphorylated substrate. Thus, for substrates that have not undergone priming phosphorylation, this is not a mechanism that prevents phosphorylation by GSK3β [140]. Furthermore, this inhibition is a competition between two phosphate groups, which can then be overcome by a higher substrate concentration [140]. Finally, phosphorylation status of S9 is in a dynamic equilibrium, i.e., it fluctuates, which means that S9 phosphorylation does not completely reduce the catalytic activity of GSK3β [140]. Similar findings that S9 phosphorylation does not affect GSK3β activity on the Wnt pathway [24,25] and that “inactive” pGSK3β-S9 form is actually the one that phosphorylates Gli3 in the Hedgehog pathway [34], also support this view. It seems that GSK3β as a part of protein complexes (axine-APC-CK1-GSK3β and Gli3-GSK3β-SuFu) is not inhibited by S9 phosphorylation. Taking the aforementioned into consideration, it appears that taking S9 phosphorylation as a fixed on/off mechanism could not be flexible enough to study the role of the enzyme in cancer development and progression. Similar view is advocated by Mitra and Roy, who suggested that the cell fate depends on GSK3β inhibitor and β-catenin concentrations, where lower inhibitor concentrations suppressed invasiveness while higher concentrations facilitated it [101]. We believe that a critical concentration of β-catenin is necessary to induce EMT.

The current evidence indicates that in spite of the significance that is given to GSK3 in ovarian cancer pathogenesis, it has not been shown useful in the prognosis of the patients with ovarian cancer. The study regarding GSK3β expression found higher survival rates among the patients with lower GSK3β expression [86], whereas the study concerning Akt kinase found higher survival rates among the patients with lower expression of inhibited pGSK3β-S9 [91]. Another study found no significant correlation between survival and GSK3β expression [5].

As a therapeutic approach, both activation and inhibition have been reported successful, but further investigations of both effects are needed, to determine more detailed pharmacokinetics of new medicines and to explain dual functions of GSK3 in cancer development. In other words, caution is necessary in order to avoid tumor progression when targeting GSK3 as it has both prooncogenic and tumor-suppressive features, especially having in mind the proposed protective functions of GSK3 against EMT [104,106,108], neoangiogenesis [110], and resistance to chemotherapy [112,113,115].

In conclusion, since the exact function of GSK3 in cancer development has not been elucidated yet, further research on the regulation and effects of this enzyme is undoubtedly needed. Although some therapeutic options targeting GSK3 exhibited promising results, GSK3 as a treatment target is still in an early phase of development. Therefore, it is essential to approach every patient individually and to keep carefully assessing possible outcomes of GSK3 focused treatment strategies.
